# Optimization of thorium solvent extraction process from feed solution with Cyanex 272 by response surface methodology (RSM)

**DOI:** 10.1038/s41598-024-66091-0

**Published:** 2024-07-02

**Authors:** F. Khanramaki, A. R. Keshtkar

**Affiliations:** Nuclear Fuel Cycle Research School, Nuclear Science and Technology Research Institute, AEOI, P.O. Box: 11365-8486, Tehran, Iran

**Keywords:** Thorium purification, Solvent extraction, Cyanex 272, Central composite design, Response surface method, Environmental sciences, Chemistry, Engineering

## Abstract

Due to the limited reserves of uranium, the abundance of thorium compared to it and other advantages, the development of the thorium fuel cycle is of interest in different countries. The optimization of thorium extraction from a feed solution produced by Saghand ore with bis(2,4,4-trimethylpentyl) phosphinic acid (Cyanex 272) on a laboratory scale was evaluated by response surface method. The operating variables include Cyanex 272 concentration of 0.001 to 0.2 mol/L, pH of 0 to 2, equilibrium time of 5 to 60 min and aqueous to organic phase ratio of 0.5 to 2.5 were conducted. The value of R^2^ = 0.9695 and an error of less than 4% indicate the validity of the model. Therefore, the model is in good agreement with the experimental results. It can be said that there are significant interactions between operational parameters, which vindicate different feedbacks of the system in different operational conditions. The results showed that the 4 mol/L sulfuric acid was a suitable agent for recovering thorium ions from the loaded organic phase. In optimum conditions, the thorium purity percentage and thorium stripping efficiency were obtained 98.99 and 94.12%, respectively.

## Introduction

Thorium is one of the radioactive chemical elements that was discovered in 1828. This element is found abundantly in nature, so that the abundance of thorium compared to uranium in the earth's crust is estimated to be 3 to 4 times higher ^1^. This element is also a byproduct the rare earth elements extraction process from monazite. In recent years, due to the development of nuclear energy, finding abundant and stable sources for nuclear fuels has gained significant importance. Therefore, thorium fuels can be considered as a suitable alternative to uranium fuels^1,2^. As a result, extraction and purification of this metal from impurities is of great importance.

Solvent extraction methods^3–6^, ion exchange^7^, liquid membrane^8^, adsorption^9,10^ and precipitation^11^ methods are among the practical methods for separation elements from different aqueous solutions. One of the most important metal extraction processes is the solvent extraction method, which is widely used in various industries such as pharmaceuticals, hydrometallurgy, and waste management. Compared to other methods, it is a very easy, low-cost method and with high potential in selectivity^12,13^. Therefore, the solvent extraction method is one of the most effective methods for metal extraction and purification in the industry.

One of the most important main components in the solvent extraction process is the choice of the extractant. Therefore, in solvent extraction systems, phosphate solvents, amines and carboxylic acids are usually studied^14^. Based on the previous studies, various compounds such as Cyanex 923^15^ (a mixture of four trialkyl phosphine oxide s), acidic compounds like bis(2-ethyl hexyl)phosphoric acid (HDEHP), bis(2-ethyl hexyl) phosphinic acid (PIA8), 2-ethyl hexyl phosphonic acid mono 2-ethyl hexyl ester (PC-88A) and tributyl phosphate (TBP) are used for extraction of thorium from different solutions^15–18^. Multi-stage separation, stripping acidity and high reagent concentration are among the limitations of using these extractants^19^. The applicability of Cyanex 272 as an extractant for thorium extraction is well established^19,20^, but research on thorium extraction from real solution is very limited. Resistance to hydrolysis, poor aqueous solubility, and the ability to fully mix with common organic diluents are among the most important advantages of Cyanex 272^21^, which can be usefully used in commercial processes for the separation of thorium, especially in nuclear applications. In a research, the solvent extraction process of thorium from nitric and sulfuric acid medium with commercial extractants such as Cyanex 272, Cyanex 302, Cyanex 301 and PC-88A in benzene as diluent has been studied^22^. Based on the analysis of the slope of the graphs, the extracted species are proposed as Th(NO_3_)_2_R_2_ and ThSO_4_R_2_. The extraction efficiency of extractants in nitric acid medium is Cyanex 272 > PC-88A > Cyanex 302 > Cyanex 301, which is reversed in sulfuric acid medium. Also, the mixture of PC-88A, Cyanex 301 and Cyanex 272 with tri-n-butyl phosphate or di-n-octyl sulfoxide resulted in significant synergism effect in thorium extraction process. Also, the behavior of thorium extraction with tributyl phosphate and bis(2,4,4-trimethylpentyl) phosphinic acid in kerosene from nitrate medium was investigated by Shari et al.^23^. The optimum conditions of variables such as equilibrium time, initial aqueous pH, concentration of TBP and Cyanex272 were 20 min, 3, 1 and 0.01 M, respectively. The mixture of TBP and Cyanex272 reveals a positive synergistic effect on the thorium extraction. The maximal synergistic enhancement coefficient is 3.86 by the mixtures of 0.008 M TBP and 0.002 Cyanex 272 extractant.

In conventional experiment by following the method of one variable at a time (OVAT), all variables are kept constant and by changing one parameter, its optimal point is obtained. Since this method cannot simultaneously examine the effect of variables on the response, it has problems, because it only considers the response to changes in individual variables^24,25^. Since the one variable method is expensive and time-consuming to optimize the effecting parameters, the surface response method is recommended to optimize the parameters. Especially when the response of a parameter is influenced by different variables, this method will be useful^26^. A number of previous studies have used the response surface method to optimize the extraction process parameters by determining exactly how each parameter affects the overall process^27–29^. In fact, the purpose of the experiment design is to obtain maximum information and knowledge about the process, by spending the minimum cost and time to carry out the process. Statistical analysis software is often used to optimize the process and analyze the results, the most famous of which is Design Expert. This software is very useful in the design of parameters and composite designs and it has the possibility of screening matrices with up to 50 different variables. Therefore, the response surface method is a proposed statistical procedure that can show the effect of the interaction of different parameters^30–34^.

Saghand mine is one of the active operational mines in Iran, which contains significant amounts of thorium along with uranium, and due to the special ore matrix, it requires the acquisition of technical knowledge for thorium extraction, which is part of the present research innovation. The main goal of the manuscript was to investigate the ability of the Cyanic 272 extractant to separate thorium from impurities from a solution with specified characteristics to reach the maximum purity of thorium. In this research, due to the high importance of thorium as an alternative fuel in the nuclear industry, the optimum conditions of thorium solvent extraction with bis(2,4,4-trimethylpentyl) phosphinic acid (Cyanex 272) as extractant from feed solution (resulting from the dissolution of 64% real thorium concentrate in nitric acid) was determined by response surface method based on the central composite design with expert design software, and the obtained results are presented as follow. Subsequent, thorium recovery from the loaded organic phase with different acidic compounds was evaluated.

## Materials and methods

### Chemical materials

Cyanex 272 provided by Merck Company and purified kerosene were used in extraction tests as extractant and diluent, respectively. Other chemical reagents in the experiments such as ammonia, nitric acid, sulfuric acid and hydrochloric acid were of analytical reagent grade.

Sagand ore leach solution was precipitated at pH = 8 with ammonia and temperature of 10 °C for 1 h. In the concentrate production stage, the values of impurities in the leaching solution were reduced as much as possible by the solvent extraction method and finally thorium concentrate was produced with a purity of 64%. The major impurities in the produced concentrate were vanadium, iron and uranium^35^. Then nitric acid 1 M was utilized to dissolve the thorium concentrate. This solution, which composition is given in Table 1, was used as feed solution in the experiments.

**Table 1:** Contents of Th, V, Fe and, U in the feed solution.

Component	In feed solution	In concentrate
	Content (g L^−1^)	% w/w
Thorium	1.25	64
Vanadium	0.40	20
Iron	0.25	13
Uranium	0.07	3

### Experimental procedure

Thorium extraction experiments from the feed solution were carried out by using Cyanex 272 and kerosene as extractant and diluent on a laboratory scale. To carry out solvent extraction process, two aqueous and organic phases (with a certain phase ratio) are transferred to a closed polyethylene container. The container is placed inside the thermostatic shaking water bath regulated by a temperature controller. After the contact time in each experiment, the aqueous and organic phases are separated by separation funnel. The concentration of metal in the aqueous phase was determined by using an inductively coupled plasma atomic emissions spectrometer (ICP-AES) manufactured by Varian, Australia, and the metal concentration in the organic phase was determined by mass balance of metal in the aqueous phase before and after the extraction processes. Also, pH was measured by using a Sartorius digital pH meter made in the United States. The extraction efficiency (E), purification factor (P), and stripping efficiency (S) are calculated by the following equations^36^:

1 $$\text{Extraction efficiency }(\text{\%E})=\frac{{[M]}_{aq, 0}-{[M]}_{aq,eq}}{{[M]}_{aq,0}}\times 100$$

2 $$\text{Purification factor }(\text{\%}P)=\frac{{\left[M\right]}_{org}}{{\left[{M}_{tot}\right]}_{org}}\times 100$$

3 $$\text{Stripping efficiency }(\text{\%S})=\frac{{[M]}_{org,0}-{[M]}_{org, eq}}{{[M]}_{org, 0}}\times 100$$

where [M]_aq,0_ and [M]_aq,eq_ represent the initial and final concentrations of the metal ions in the aqueous phase. [M]_org_ and [M_tot_]_org_ respectively represent the final concentrations of metal ions and the concentration of total metals in the organic phase. [M]_org,0_ and [M]_org,eq_ demonstrate the initial and final concentrations of the metal ions in the organic phases, respectively.

### Experimental design and statistical analysis

The central composite design (CCD) is a method that can be efficiently applied to develop the second-order response model with few numbers of factors n (2 ≤ n ≤ 6). Applying experimental design, a quadratic approximation based on CCD is frequently utilized to develop a response surface model. The standard and complete quadratic model for k variables contains (k + 1)(k + 2)/2 parameters in the experiment design software is as follows^37^:

4 $$y={\eta }_{0}+\sum_{i=1}^{k}{\eta }_{i}{x}_{i}+\sum_{i=1}^{k}{\eta }_{ii}{{x}_{i}}^{2}+\sum_{i=1}^{k}{\eta }_{ij}{x}_{i}{x}_{j}+\sigma$$

where η_0_, η_i_, η_ii_, η_ij_, x_i_, k, and σ represent the constant term, the coefficient of the linear parameter, the coefficient of the quadratic parameter, the coefficients of the interaction parameters, the variables, the variables' number, and the residual related to the runs, respectively. Each variable in the model has a coefficient. At standardized model, numerical magnitude of coefficients indicates the importance of that parameter in the modeled response. Also, the inverse effect of the relevant factor on the modeling response is shown with a negative coefficient.

The influence of each variable and their interaction was investigated using the Design-expert program. Significant expressions (P-value < 0) in the model were determined using analysis of variance (ANOVA) for each answer. Statistically insignificant terms (P-value > 0.05) were removed from the model. Also, the experimental data were fitted to produce the final model. The response surface plots, derived from the selected model, was used to examine the interaction of the variables^38^.

ANOVA has been utilized in order to verify the relevance of the second-order models. Analysis of variance (ANOVA) establishes the generated quadratic model's significance. Each term's significance is estimated using the ANOVA analysis and the F-test. The large F-value shows that most of the variation in the output can be explained by the developed regression equation. In order to determine whether F is large enough to demonstrate statistical significance, the corresponding p-value is also utilized. The developed model and the terms are statistically significant if the p-values are less than 0.05^39–41^. The F-value, a measurement of data variance about the mean based on the ratio of mean square of group variance due to error, was applied to evaluate the statistical significance of the quadratic models. If the calculated F value be higher than the tabulated F value, it indicates that the model accurately predicts the experimental data^42^.

First, the single-variable method was used to optimize the contact time of the two phases in order to maximum thorium purification. Then, the statistical analysis software Design Expert 12 was used to design the experiments and optimize other effective process parameters. In this research, three independent process parameters (such as Cyanex 272 concentration, pH and phase ratio) were evaluated using response surface method based on central composite design with experiment design software at five levels.

Every process variable in a CCD design is examined at five different levels (− α, − 1, 0, + 1, + α); each of these values represents an original variable value. Using a simple linear transformation, coding of variable levels is done so that + 1 is attributed to the highest value and -1 to the lowest value of the main variable. The average of these two values is assigned to 0 While the maximum and minimum values are applied with + α and − α. The values of α depend on the number of variables under study, so that for two, three and four variables it is 1.41, 1.68 and 2.00, respectively^38^. In the current research, the independent variables and levels were performed according to Table 2.

**Table 2:** The variables and levels used for RSM method.

Factors	Symbols	Level				
		− α	− 1	0	+ 1	+ α
Cyanex 272 concentration (mol/L)	A	0.001	0.05	0.1	0.15	0.2
pH	B	0	0.5	1	1.5	2
Aqueous to organic phase ratio-A/O	C	0.5	1	1.5	2	2.5

RSM was used to maximize the thorium purification in extraction process. Also, regression analysis was used to compare the performance of the answers obtained on the laboratory data. The statistical testing is done by ANOVA analysis with P-Value, F-Value, R-Squared, adjusted R-Squared and predicted R-Squared^31^. The operating conditions as suggested by Central Composite Design (CCD) and the obtained responses for purification of thorium at each run are reported in Table 3. In the following, three-dimensional diagrams of effective parameters are drawn using Design Expert software.

**Table 3:** The studied variables levels in each run with experimental response based on CCD method.

No.	A: [Cyanex 272], (M)	B: pH	C: A/O	Thorium purity (%)	
				Experimental	Predicted
1	0.15	1.50	1.00	88.63	88.55
2	0.05	0.50	2.00	98.10	97.30
3	0.10	1.00	1.50	97.14	96.26
4	0.05	1.50	1.00	93.00	92.16
5	0.15	1.50	2.00	92.47	91.43
6	0.15	0.50	2.00	96.83	96.80
7	0.15	0.50	1.00	98.81	99.68
8	0.10	1.00	2.50	97.26	98.58
9	0.10	1.00	1.50	95.87	96.26
10	0.10	1.00	1.50	96.12	96.26
11	0.10	1.00	1.50	95.85	96.26
12	0.001	1.00	1.50	97.61	98.81
13	0.10	1.00	0.50	98.01	97.55
14	0.10	1.00	1.50	95.87	96.26
15	0.10	1.00	1.50	95.88	96.26
16	0.10	0.00	1.50	96.23	95.66
17	0.10	2.00	1.50	81.86	83.31
18	0.05	1.50	2.00	97.79	96.07
19	0.05	0.50	1.00	98.99	99.14
20	0.20	1.00	1.50	95.02	94.69

## Results and discussion

### Optimization of contact time

**Figure 1:**
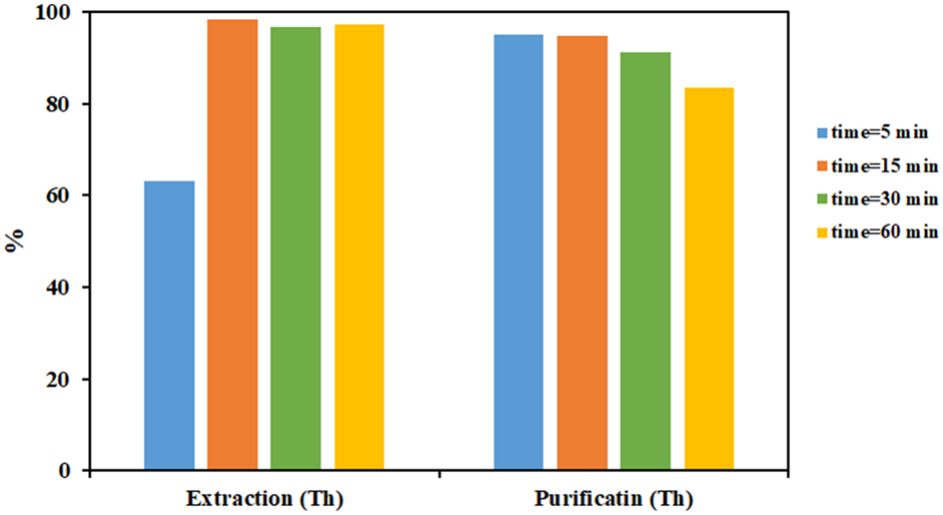
Investigating effect of the contact time on the thorium extraction efficiency and thorium purity percentage (experimental conditions: [Cyanex 272] = 0.1 M in kerosene, PH = 1, A/O = 1, T = 25 °C).

First, the optimization of the contact time of two phases was investigated as one of the process parameters of thorium extraction from the feed solution with Cyanex 272. Table 4 shows the results of investigation the effect of the contact time on the extraction efficiency of elements and the purity of thorium in the loaded organic phase. In the following, the changes in the thorium extraction efficiency and thorium purity percentage in terms of time by the single variable method are given in Fig. 1 as clearly.

**Table 4:** Influence of the contact time on the extraction efficiency of the elements and thorium purity percentage (experimental conditions: [Cyanex 272] = 0.1 M in kerosene, PH = 1, A/O = 1, T = 25 °C).

Contact time (min)	Extraction efficiency (%)				Purity (%)
	Thorium	Vanadium	Iron	Uranium	Thorium
5	63.18	8.37	2.16	40.92	95.09
15	98.33	12.27	4.86	74.6	94.89
30	96.81	12.32	5.41	83.58	91.66
60	97.18	16.75	6.13	90.96	88.34

The results showed that by increasing the contact time from 5 to 15 min, the thorium extraction efficiency increased significantly. But by increasing the contact time to more than 15 min, no significant change was observed in the thorium extraction efficiency. On the other hand, it can be seen that by increasing the contact time of two phases, the extraction efficiency of other elements increases. This leads to a decrease in the thorium purity in the loaded organic phase. This is an unfavorable effect for having a product with high purity. Therefore, according to the obtained results, the contact time of 15 min was chosen as the optimum time to have a higher purity of thorium in the experiments.

### Single variable parametric studies

In this section, the effects of process parameters, including the Cyanex 272 concentration, pH, and aqueous to organic phase ratio, on the thorium purity percentage in the loaded organic phase after the extraction process, were examined. The results obtained from the experimental design using the response surface method for this parameter are presented. Thorium purity percentage in each experiment is calculated by Eq. (2). The final experimental model in terms of actual parameters was determined and was proposed as Eq. (5) by the experiment design software, where the empirical coefficients being dependent on the values of regression coefficients and on the operating range of each operating variable. The significance of each regression coefficient was determined by applying the Student t-test^42^. The relationship presented in order to determine the thorium purity percentage based on actual process parameters were obtained with Design Expert software as Eq. (5):

5 $$\begin{aligned} Thorium \;\; purity \left(\%\right) & =104.41+26.68 A+2.90 B-9.60 C-41.43 \left(A\times B\right) \\ & \quad -10.33 \left(A\times C\right)+5.75 \left(B\times C\right)+48.44 \left({A}^{2}\right)-6.78 ({B}^{2})+1.80 ({C}^{2}) \end{aligned}$$

In the above equation, A, B, and C is Cyanex 272 concentration, pH of the aqueous phase and aqueous to organic phase ratio, respectively. The increasing and decreasing trend of a parameter in the above equation is indicated by positive and negative signs. The results of the analysis of variance and accuracy of the model (ANOVA) for the thorium purity percentage is presented in Table 5.

**Table 5:** Variance analysis of regression equations on thorium purity percentage.

Source	Sum of squares	df	Mean square	F value	p-value
Model (quadratic)	295.49	9	32.83	35.35	< 0.0001
A-[Cyanex 272]	12.83	1	12.83	13.81	0.0040
B-pH	166.38	1	166.38	179.13	< 0.0001
C-A/O	1.02	1	1.02	1.10	0.3187
AB	4.88	1	4.88	5.25	0.0449
AC	0.53	1	0.53	0.57	0.4683
BC	16.55	1	16.55	17.81	0.0018
A^2^	0.24	1	0.24	0.26	0.6202
B^2^	74.15	1	74.15	79.84	< 0.0001
C^2^	4.37	1	4.37	4.71	0.0552
Residual	9.29	10	0.93		
Lack of fit	8.00	5	1.60	6.22	0.331
Pure error	1.29	5	0.26		
Cor total	304.78	19			

**Figure 2:**
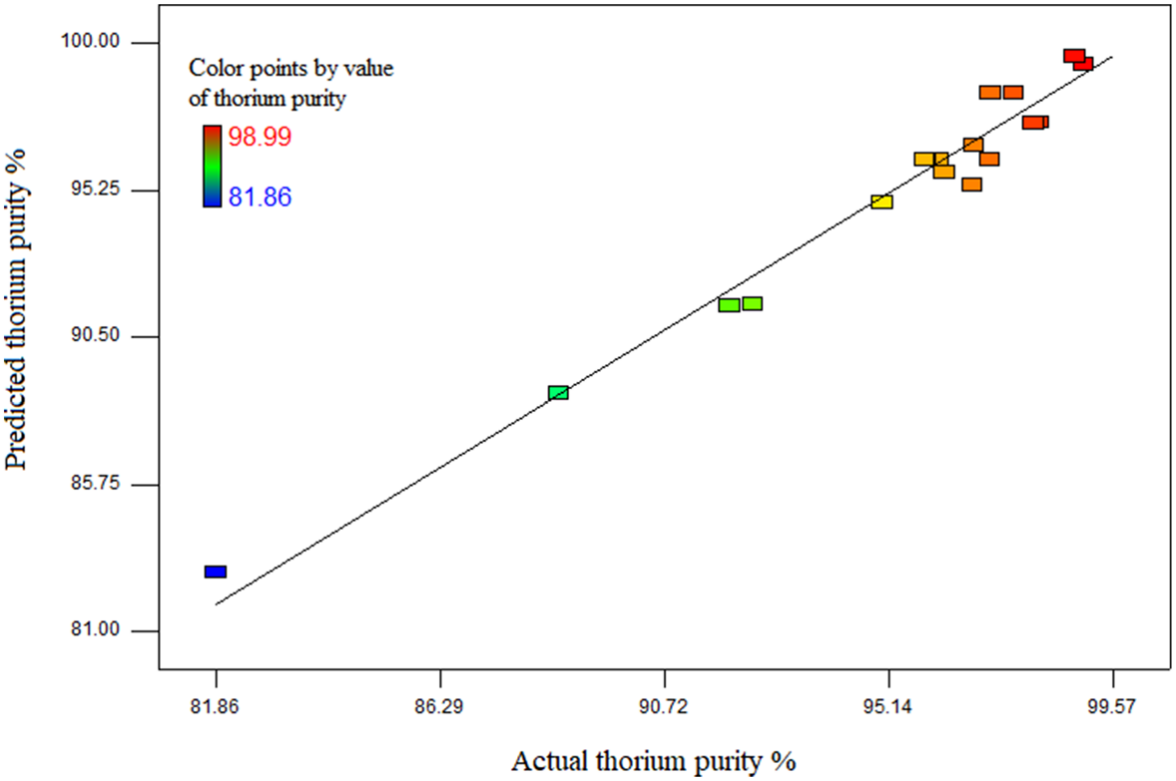
The consistency of the experimental data with the predicted data by the equation of the model.

The results of the analysis of variance showed that the quadratic model is the best model. The F-value ~ 35.35 and lack of fit for F-value ~ 6.22 are the meaningful and significant data. On the other hand, the following values are obtained for this model R^2^ = 0.9695 and adj. R^2^ = 0.9421, while the "p-value" term can be considered significant at p < 0.05. Therefore, considering these values, it can be said that the proposed model by the software has a considerable accuracy in fitting the data. Based on the values for the p-value in Table 4, it can be concluded that the liner terms (Cyanex 272 concentration, pH) and quadratic term (pH^2^) have a significant influence on the thorium purity percentage. The low p value related to the interaction of pH and phase ratio parameters (BC) (equal to 0.0018) indicates the significant effect of this term. On the other hand, two other interactions (AB and AC) had a negligible effect with p-value equal to 0.0449 and 0.4683, respectively. Therefore, these expressions can be removed from the equation of the model. Figure 2 shows the relationship between experimental data values and data values predicted by the model equation. According to the diagram, it can be said that the quadratic model is able to making suitable response predictions.

#### Effect of Cyanex 272 concentration

**Figure 3:**
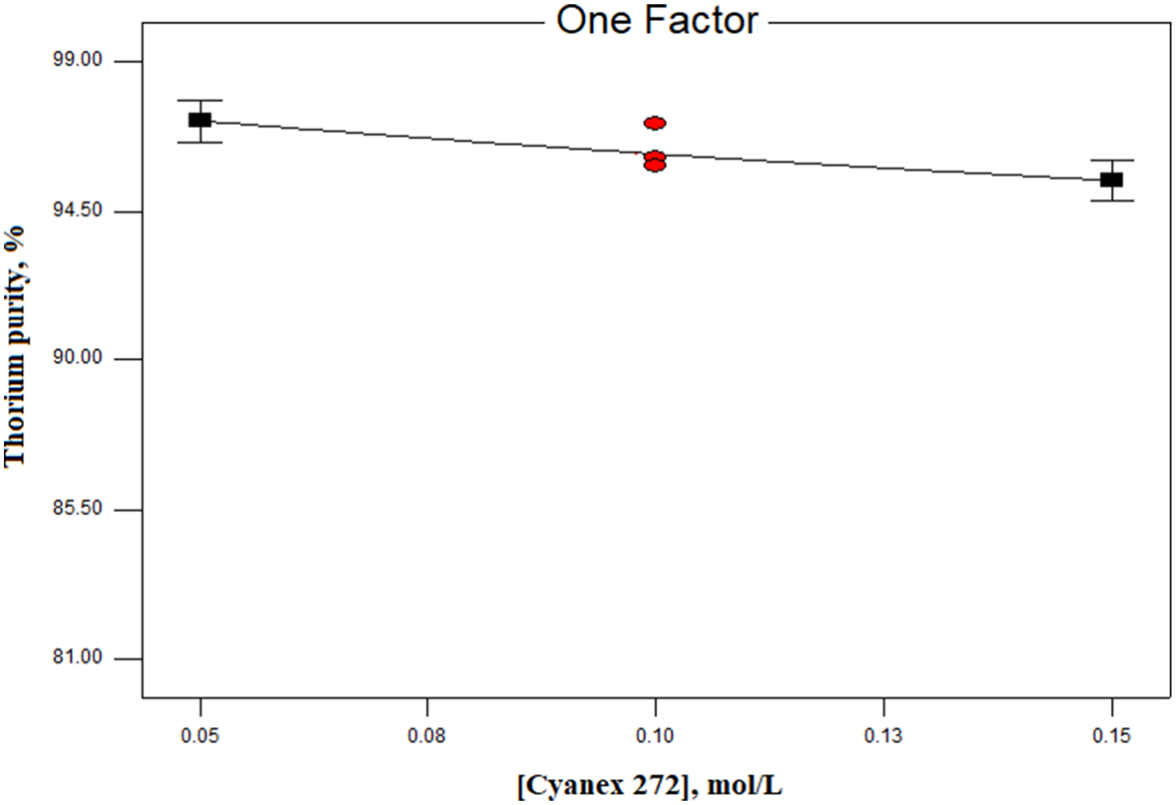
The effect of Cyanex 272 concentration on thorium purity percentage (experimental conditions: kerosene as diluent, pH = 1, A/O ratio = 1.5, equilibrium time = 15 min, T = 25 °C).

One of the important process parameters in extraction section is the initial concentration of the extractant, where the maximum possible metal extraction occurs. By increasing the Cyanex 272 concentration, the metal extraction efficiency increases. Therefore, with an increase in the Cyanex 272 concentration, the thorium extraction and the impurities present in the thorium nitrate solution also increase, leading to a decrease in thorium purity in the loaded organic phase. Therefore, the Cyanex 272 concentration variations on the thorium purity were investigated, and the results obtained are presented in Fig. 3. According to this diagram, it was observed that with an increase in the Cyanex 272 concentration from 0.05 to 0.15 M, the thorium purity percentage decreases. Therefore, in the present research, the optimal concentration of Cyanex 272 to have high thorium purity was considered to be 0.05 mol/L.

#### Effect of pH

**Figure 4:**
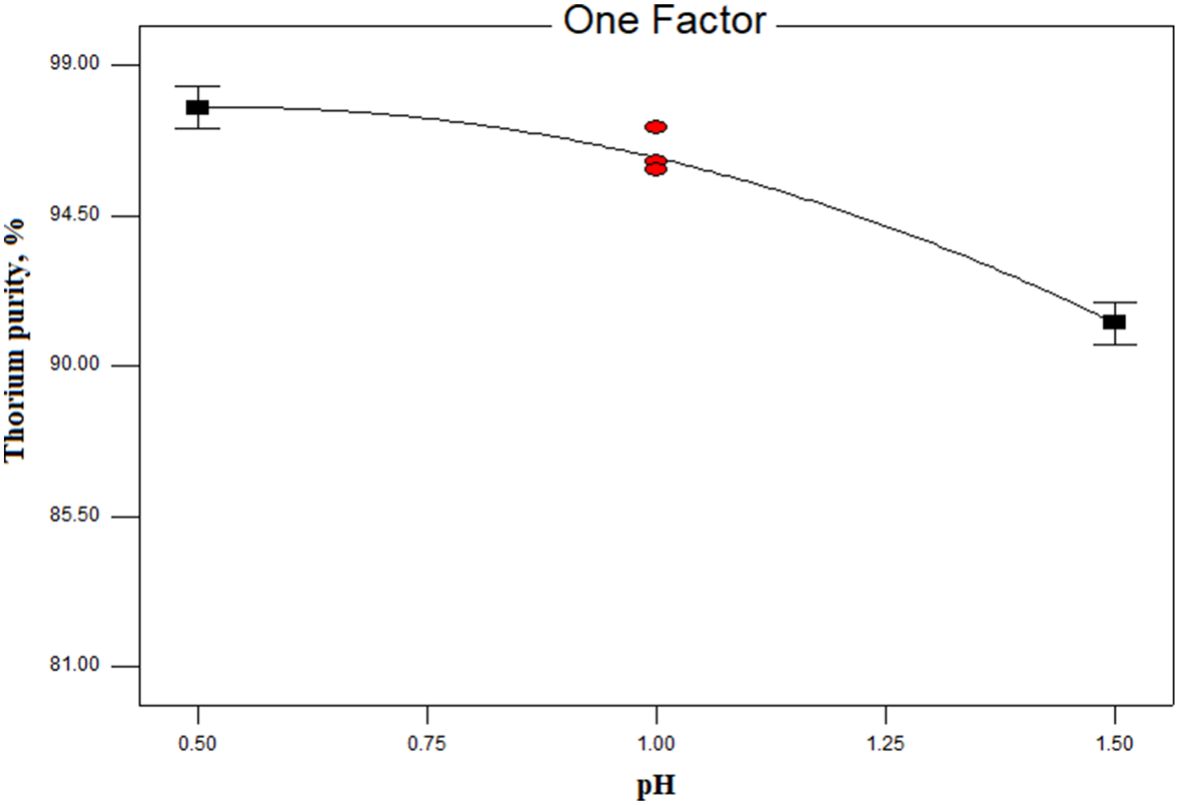
The effect of pH on the thorium purity percentage (Experimental Conditions: [Cyanex 272] = 0.1 mol/L in kerosene, A/O ratio = 1.5, equilibrium time = 15 min, T = 25 °C).

The effect of the pH of the aqueous phase on the thorium purity was examined. The changes in the pH of the aqueous phase (in the range of 0–2) on the thorium purity are shown in Fig. 4. The results of the variations in the initial pH of the aqueous phase on the thorium purity percentage indicated that by reducing the acidity of the feed solution (pH), the purity of thorium decreases. So that the highest purity of thorium was obtained at a pH equal to 0.5.

#### Effect of the aqueous to organic phase ratio

**Figure 5:**
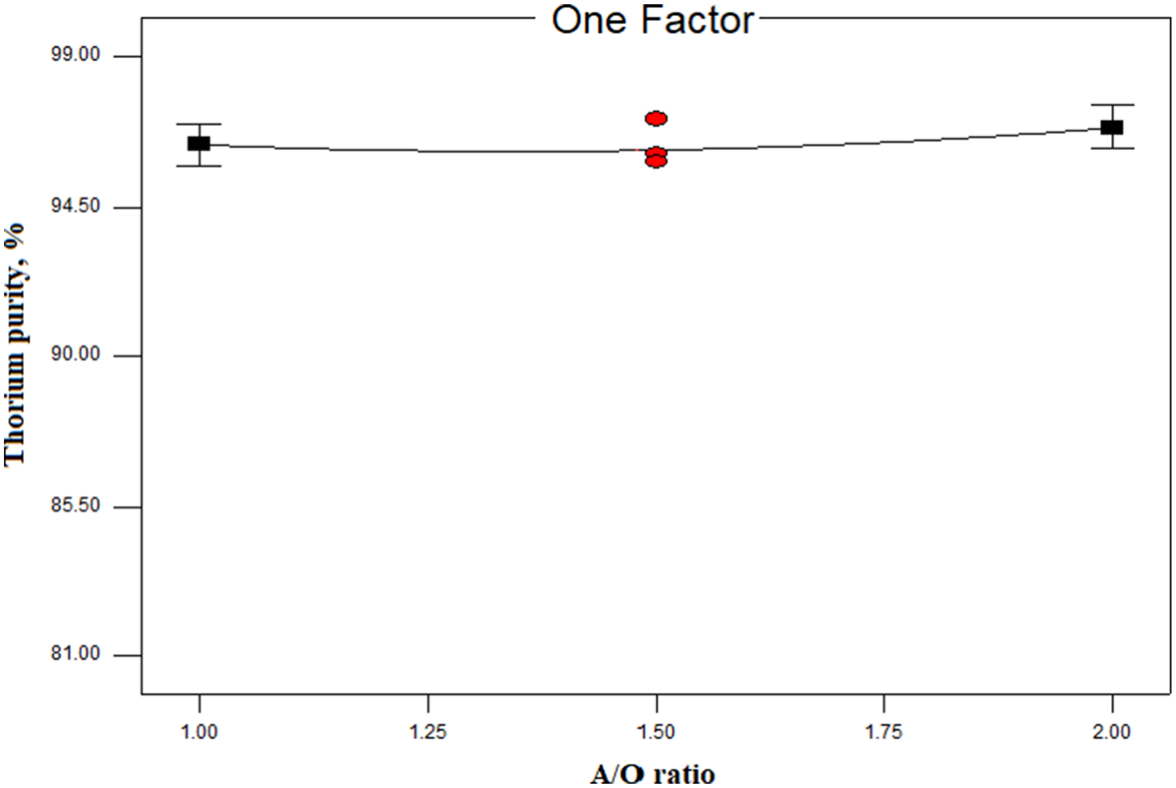
Investigation of the effect the phases ratio (A/O) on thorium purity percentage (experimental conditions: [Cyanex 272] = 0.1 mol/L in kerosene, pH = 1, equilibrium time = 15 min, T = 25 °C).

The results of the variations in the phases ratio (A/O) on the thorium purity percentage are presented in Fig. 5. According to this graph, it was observed that the A/O phase ratio did not have a significant impact on the thorium purity percentage.

### Optimization of the thorium purity by RSM method

#### Investigation the interaction effect of process parameters on the thorium purity

**Figure 6:**
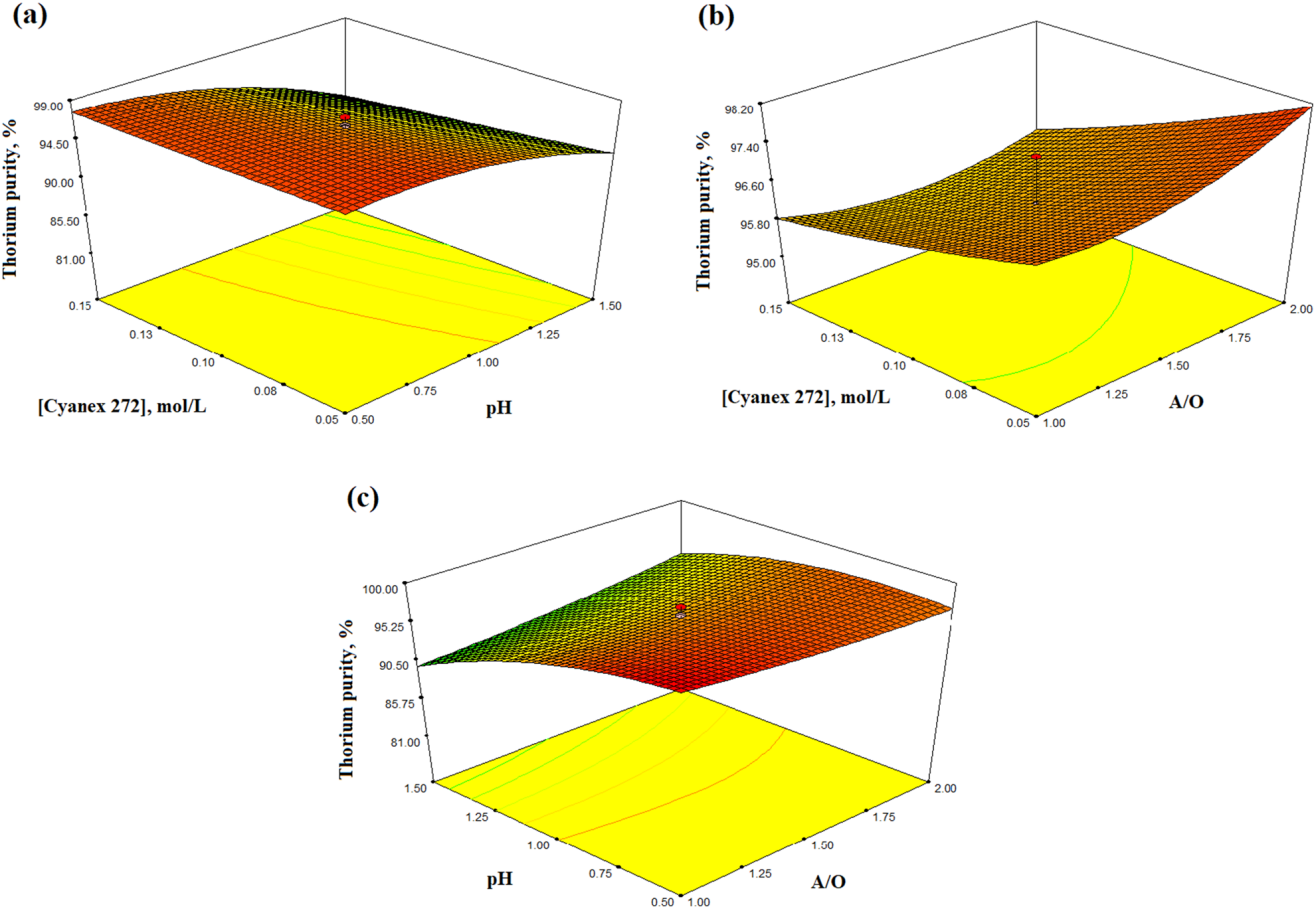
Response surface plot of thorium purity as a function of the process parameters.

The interaction effect of process parameters on the thorium purity percentage has been represented as three dimensional plots in Fig. 6. Based on the p-values in Table 4 for interaction terms, it can be concluded that the Cyanex 272 concentration and the pH, as well as the phase ratio and the pH, have significant effects on the thorium purity percentage. The interaction effect of Cyanex 272 concentration and phase ratio has a small effect, which is not significant.

Examining Fig. 6a, it can be inferred that at a constant phase ratio (A/O = 1.5), increasing the Cyanex 272 concentration within the range of 0.05 to 0.15 M and decreasing the pH within the range of 0.5–1.5, leads to an increase in the thorium purity percentage from the feed solution. Considering Fig. 6b, it can be concluded that at a constant pH (pH = 1), reducing the concentration of the Cyanex 272 within the range of 0.05–0.15 M and increasing the phase ratio within the range of 1–2, leads to an increase in thorium purity percentage in the loaded organic phase. Also, according to Fig. 6c, it can be concluded that at a constant of Cyanex 272 concentration (0.1 mol/L), reducing the pH within the range of 0.5–1.5 and decreasing the phase ratio within the range of 1–2, leads to an increase in thorium purity percentage in the loaded organic phase.

#### Validation of the presented models for the thorium purity

A quadratic model (Eq. 5) was proposed to investigate the effect of process parameters on thorium purity. In Table 6, the validation results of the model are reported. The results of variance analysis showed that the model fits the data well within the defined range. With some new random examinations, the accuracy of the model was evaluated. The error of the values predicted by the presented model is calculated by the following equation:

**Table 6:** Error determination of the predicted values by the model and experimental values for thorium purity.

No	Process parameters			Thorium purity (%)		Error (%)
	[Cyanex 272], (mol/L)	pH	A/O	Experimental	Predicted	
1	0.09	0.77	1.42	95.73	97.51	1.86
2	0.06	0.59	1.69	95.11	97.72	2.74
3	0.07	0.9	1.85	93.84	97.66	4.07
Opt	0.05	0.5	1.00	98.99	99.14	0.15

6 $$Error(\%)=\left|\frac{Experimental \; data-Predicted \; data}{Experimental \; data}\right|\times 100$$

Based on the results, it can be concluded that the model offered by the software has an error of less than 4%. Therefore, the model is in good agreement with the experimental results.

### Extraction isotherm (McCabe–Thiele diagram)

**Figure 7:**
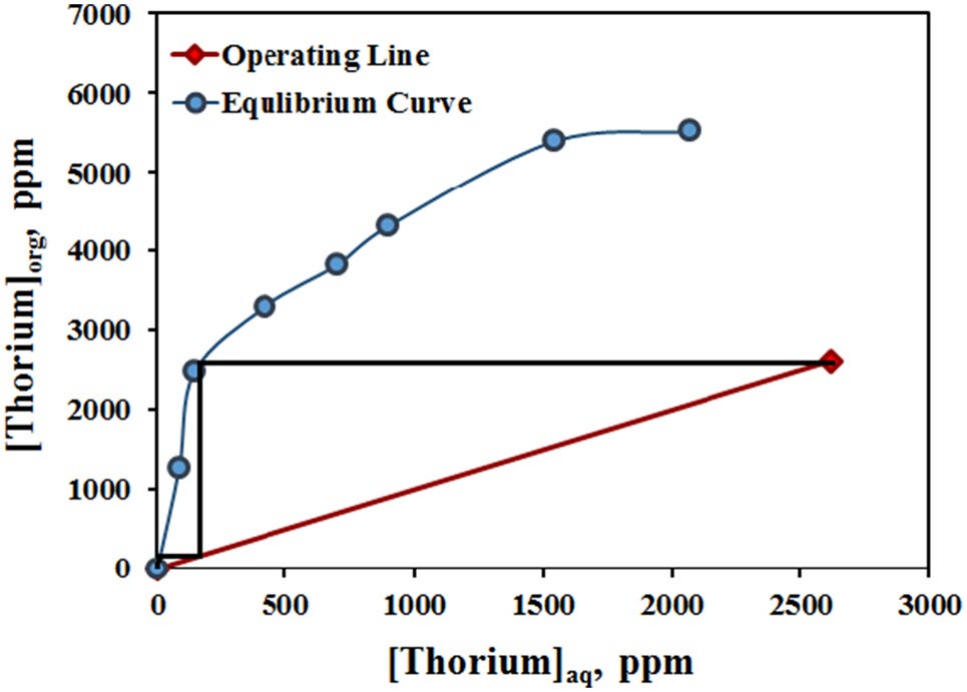
Thorium extraction distribution isotherm and McCabe Thiele diagram (experimental conditions: [Cyanex 272] = 0.1 mol/L in kerosene, pH = 1, equilibrium time = 15 min, T = 25 °C).

The McCabe–Thiele diagram of thorium extraction from feed solution was drawn with Cyanex 272 as extractant in optimal process conditions with different phase ratios at 25 °C, as Fig. 7. Also, this diagram was completed to estimate the number of theoretical extraction steps required using an optimized organic system with an phases ratio equal to 1as operating line. According to the McCabe diagram, it can be said that in optimal conditions with two equilibrium stages, thorium extraction efficiency will reach 99.9%.

### Stripping process investigation

After the solvent extraction process with Cyanex 272 as extractant, the loading organic (LO) phase was used for stripping study to separate the maximum value of thorium with minim quantity of other elements. To investigate the thorium recovery from the LO phase, different acidic solutions such as sulfuric acid (H_2_SO_4_), hydrochloric acid (HCl), and nitric acid (HNO_3_) with a concentration of 0.1 mol/L were examined. The results of these experiments are presented in Table 7.

**Table 7:** Investigation of the thorium recovery with different acid solutions (experimental Conditions: O/A = 1, equilibrium time = 30 min, T = 25 °C).

Element	HCl (0.1 mol/L)	HNO_3_ (0.1 mol/L)	H_2_SO_4_ (0.1 mol/L)
	Stripping efficiency (%)	Stripping efficiency (%)	Stripping efficiency (%)
Thorium	1.50	0.22	38.42
Vanadium	21.61	53.44	3.12
Iron	56.34	67.11	3.50
Uranium	34.82	0.44	15.33

The results indicate that sulfuric acid is a more suitable solution for thorium recovery from the organic phase containing Cyanex 272 in kerosene. According to the observed results, a sulfuric acid solution with a concentration of 0.1 mol/L can recover approximately 38% of thorium from the LO phase in a single step. Therefore, to increase the thorium recovery from the LO phase, variations in the acid concentration were evaluated, and the results are presented in Table 8.

**Table 8:** Investigating thorium recovery from organic phase with different concentrations of sulfuric acid solution (experimental conditions: O/A = 1, equilibrium time = 30 min, T = 25 °C).

Element	H_2_SO_4_(2 mol/L)	H_2_SO_4_(3 mol/L)	H_2_SO_4_(4 mol/L)
	Stripping efficiency (%)	Stripping efficiency (%)	Stripping efficiency (%)
Thorium	68.04	75.10	94.12
Vanadium	11.95	19.46	20.36
Iron	6.77	8.35	9.12
Uranium	33.31	47.74	50.39

Based on the observed results, it can be seen that a sulfuric acid solution with a concentration of 4 mol/L can recover approximately 94% of thorium from the LO phase in a single step. Considering the presence of impurities, this level of recovery is acceptable. To achieve 100% thorium recovery, it is possible to perform at least one more stage of recovery using a sulfuric acid solution with a concentration of 4 mol/L.

## Conclusions

In this article, the purification of thorium from feed solution in the presence of vanadium, iron and uranium impurities was evaluated with Cyanex 272 as extractant. First, the optimization of the contact time of the two phases was carried out using a one variable method. Then, in order to optimize other effective process parameters such as Cyanex 272 concentration (A), pH (B) and phase ratio (C), the design software by using surface response method at five different levels was used. The variance analysis of the fitting model was performed. The value of R^2^ equal to 0.9795 shows the significant accuracy of the model proposed by the software. Also, based on the p-value, it can be concluded that the process parameters including the concentration of Cyanex 272, the pH and the interaction effect of the Cyanex 272 concentration and the pH and the pH and the aqueous to the organic phase ratio have a significant effect on the thorium purity percentage. On the other hand, the phase ratio and the interaction effect of Cyanex 272 concentration and the phase ratio do not have a significant effect on the thorium purity percentage. The operating parameters such as the contact time, Cyanex 272 concentration, pH and phase ratio (A/O) were optimized 15 min, 0.05 mol/L, 0.5 and 1, respectively. The suitable type and concentration of stripper for striping thorium from loaded organic phase were obtained H_2_SO_4_ with a concentration of 4 mol/L. The purity percentage of thorium and the separation efficiency of thorium from the loaded organic phase in optimal conditions have been reported as 98.99 and 94.12%, respectively. Also, by drawing the McCabe diagram for the thorium extraction process, it can be concluded that thorium extraction efficiency reaches 99% with two equilibrium stages.

## Data Availability

The datasets used and/or analyzed during the current study are available from the corresponding author on reasonable request.
